# Visual evoked potentials among Indian patients with optic nerve disorders

**DOI:** 10.6026/9732063002001266

**Published:** 2024-10-31

**Authors:** Ruchi Kothari, Sai Shanmukh Vemparala, Siraj Muhammed Shafy, Mayur Wanjari, Labdhi Sangoi, Ravi Sangoi

**Affiliations:** 1 Department of Physiology, Mahatma Gandhi Institute of Medical Sciences, Sevagram, Wardha, Maharashtra, India; 2 Department of Physiology, Andhra Medical College, Medical College Road King George Hospital, opp. Collector Office, Maharani Peta, Visakhapatnam, Andhra Pradesh, India; 3 Department of Medicine, Jawaharlal Nehru Medical College, Belgaum, KLE University Campus, Nehru Nagar, Belagavi, Karnataka, India; 4 Department of Research, Datta Meghe Institute of Higher Education & Research (DMIHER), Sawangi, Maharashtra, India; 5 Deprtment of Research, Government Medical College , Jalna, Maharashtra; 6 Depertment of Internal Medicine, Government Medical College and General Hospital, Baramati, Pune, Maharashtra, India

**Keywords:** Optic nerve, visual evoked potentials, case control, visual impairments, diagnosis

## Abstract

Optic nerve disorders comprise a large part of causes of visual impairments and can be associated with various clinical presentations. Accurate diagnosis and treatment sometimes would be achieved by using advanced techniques in diagnostic approaches such as Visual Evoked Potentials (VEP). This study therefore profiles optic nerve disorders and estimates the role VEP plays in the diagnosis and prognosis of these conditions in a rural tertiary care hospital. Hundred cases diagnosed with optic nerve disorders and 200 controls matched for age and gender were recruited from the Eye OPD. Structured questionnaires, clinical examination and VEP testing were done to retrieve information. Analysis by SPSS version 23 was carried out. In the study, the most common disorders found were optic neuritis, followed by ischemic optic neuropathy, and then glaucoma. VEP revealed statistical abnormalities concerning P100 latency and amplitude in the patients with optic nerve disorders as compared to controls. P100 latency was significantly prolonged with a mean comparison of p<0.001, while the amplitude was reduced with p<0.001 when compared to the controls. VEP is an important diagnostic and prognostic tool in optic nerve diseases. It would tend to provide objective and reproducible data, thus helpful for early detection and management.

## Background:

Optic nerve disorders belong to the heterogeneous group of conditions and have caused severe visual impairment and disability [1]. These problems affect the optic nerve, which carries visual information from the retina to the brain and may cause a large range of possible visual deficits depending on location and extent of injury [2]. Some of the common causes of optic nerve dysfunction include optic neuritis, ischemic optic neuropathy, glaucoma, and compressive lesions [3]. The lack of advanced imaging and diagnostic modalities compound the problems of diagnosis in a rural tertiary care setting. The VEP has come up as a non-invasive cost-effective diagnostic tool which may provide information on the functional integrity of visual pathways [4]. VEP measures the electrical activity of the visual cortex after a visual stimulus and is considered an objective measure of the function of the visual pathway. It is helpful in an indeterminate clinical presentation or structural imaging that does not strongly support a particular diagnosis [5]. Although potential, the use of VEP remains mainly limited to rural areas. Besides, data regarding its utility in the diagnosis of different types of optic nerve disorders is very limited [6]. Therefore, it is of interest to bridge the gap by profiling the optic nerve disorders presenting in the Eye OPD of a rural tertiary care hospital and assessing the role of VEP in their diagnosis and prognosis [7].

## Methodology:

A cross-sectional observational study over one-year period, from 1st October 2015 to 1st October 2016, conducted at Mahatma Gandhi Institute of Medical Sciences, Sevagram and Wardha. Therefore, it is of interest to observe the profile of optic nerve disorders and assess the role of VEP in these conditions.

## Study population:

There were 100 cases that had been diagnosed with optic nerve disorders. There were 200 age- and gender-matched controls without any known optic nerve or visual pathway disorders. Cases were recruited consecutively from the Eye OPD, and controls were selected from the general population attending the hospital for routine check-ups.

## Inclusion criteria:

[1] Patients of any age diagnosed with optic nerve disorders such as optic neuritis, ischemic optic neuropathy, glaucoma and hereditary optic neuropathies.

[2] Patients who could cooperate for VEP and ocular examination.

## Exclusion criteria:

[1] Patients with lens or corneal opacities, miotic pupil, or recent use of eye medications (mydriatics or cycloplegics in the past 12 hours).

[2] Patients with systemic conditions affecting the performance of VEP, neurological disorders, or unwilling to participate.

[3] Uncooperative or febrile patients.

## Data collection:

Data were collected using structured questionnaires covering demographic details, clinical history and physical examination findings. Detailed ocular examination, including pupil reactions, anterior and posterior segment examination, was performed for all participants.

## Visual evoked potentials (VEP) testing:

VEP testing was conducted using the transient pattern reversal method with a black-and-white checkerboard pattern displayed on a VEP monitor. The parameters for VEP testing were as follows:

## Stimulus configuration:

1.7 Hz pattern reversal rate, 8x8 check size, 59 cd/m^2^ luminance and 80% contrast level.

## Electrode placement:

According to the 10-20 International System, the reference electrode was placed at Fz, the ground electrode at CZ and the active electrode at Oz.

## Recording conditions:

The recordings were made in a quiet, darkened room with the participant seated 1 meter from the screen. The recording was done monocularly with corrective glasses if necessary.

## Study parameters:

## P100 Latency:

Time interval between the onset of the visual stimulus and the first maximum positive deflection

## P100 Amplitude:

Measured from the peak of N70 to the trough of P100

## P100 Duration:

Time between the peaks of N70 and N155 waves

## N70 and N155 Latencies:

Time intervals between the onset of the visual stimulus and the first and second negative waves, respectively

## Statistical analysis:

Data were entered into a spread sheet and analyzed using SPSS version 23. Continuous variables were expressed as mean ± standard deviation and compared using the independent t-test. Categorical variables were compared using the chi-square test. A p-value <0.05 was considered statistically significant.

## Results:

Table 1 shows demographic characters of the study participants. Table 2 show types of optic nerve disorders in the participants. Table 3 shows VEP parameters in the cases and the control. Table 4 shows the visual evoked potential abnormalities. Table 5 shows impact of VEP findings. Table 6 shows comparison of VEP parameters by age. Table 7 shows VEP abnormalities in different genders. Table 8 shows duration of symptoms and VEP findings. Table 9 shows predictive value of VEP. Table 10 shows cost effectiveness of VEP in diagnostic management.

There was no significant difference in the demographic characteristics between the case and control groups, indicating successful matching. Optic neuritis was the most common disorder, followed by ischemic optic neuropathy and glaucoma. There were significant abnormalities in VEP parameters in the case group compared to controls, indicating impaired visual pathway function in patients with optic nerve disorders. Optic neuritis had the highest proportion of VEP abnormalities, followed by ischemic optic neuropathy and hereditary optic neuropathy. VEP played a crucial role in confirming the diagnosis in 80% of cases and led to changes in the treatment plan for 50% of patients. It also facilitated follow-up and monitoring in 70% of cases, indicating its utility in the on-going management of optic nerve disorders. P100 latency was significantly prolonged, and amplitude reduced in older age groups compared to younger individuals, indicating age-related decline in optic nerve function. Males exhibited a higher percentage of prolonged P100 latency and reduced amplitude compared to females, suggesting potential gender differences in the impact of optic nerve disorders on visual pathway function. Data shows that longer the duration of symptoms, the higher the percentage of VEP abnormalities, indicating the progressive impact of optic nerve disorders on visual function over time. VEP showed high sensitivity and specificity in differentiating various optic nerve disorders, particularly optic neuritis, demonstrating its diagnostic utility in a clinical setting. VEP proved to be very cost-effective in diagnosing and managing optic nerve disorders. This method is much less expensive in terms of diagnosis and treatment as compared with more traditional methods. It also cut the time to diagnosis and treatment and generally patients were a lot more satisfied with the overall process than when other, more conventional methods were used.

## Discussion:

This study substantiates the excellent value of VEP in diagnosing and managing optic nerve pathology within a tertiary care centre in the rural setting [8]. VEP proved to be highly sensitive and specific for differentiating most of the optic nerve disorders, particularly optic neuritis and ischemic optic neuropathy that constituted the most common condition seen during the study [9]. The significant abnormalities in VEP parameters, including prolongation of latency and reduction of amplitude of P100, in patients compared to controls, suggest that VEP may be useful for the assessment of the functional integrity of the visual pathways [10]. These findings are compatible with other studies that draw attention to the relevance of VEP in the diagnosis and follow-up of optic nerve disorders [11]. Such findings also establish the fact that VEP is greatly useful in conditions where clinical presentation may be ambiguous or when structural imaging has failed to come up with conclusive results [12]. It has even aided in changes to treatment for 50% of the patients while it is also able to monitor the other 70% of the cases. The ability of VEP to provide objective quantification of the dysfunction in the visual pathway makes it an important adjunct in the comprehensive evaluation of optic nerve disorder [13].

In addition, it was demonstrated that VEP is economic in terms of lower diagnostic and treatment costs and diagnosis with the consequent treatment achieved at an earlier time than other traditional techniques [14]. This is more crucial in resource-limited rural settings where advanced imaging modalities are not available [15]. The study also found that both age and the duration of symptoms at presentation had significant effects on VEP outcomes. Increased age and the duration of symptoms were correlated with higher degrees of abnormalities in VEP, indicating that optic nerve damage progressively occurred in a more chronic manner over time. In summary, it emphasizes the importance of early diagnosis and intervention to prevent permanent loss of vision. Although the study has many strong points, there are still some limitations. The cross-sectional design will limit the study of long-term outcomes, and therefore, the findings of this study may not be extended to the general population because it was a single-centre setting. To confirm the results of this study and to establish whether or not the predictive value of VEP in other optic nerve disease, further studies with higher numbers and longitudinal follow-up are necessary. Future studies can be done which brings out the high predictive value of both positive and negative VEPs as suggested by previous study [16].

## Conclusion:

VEP offer objective, reproducible data at relatively a lower cost on the functioning of the visual pathway as a tool in optic nerve disorders diagnostics. They help in managing and following optic nerve disorders, more especially in resource-poor settings. Its prompt use by clinicians might enhance its diagnostic accuracy, enhance treatment outcomes and reduce healthcare costs. Further studies are warranted to elucidate its long-term prognostic value and expand its use in varying clinical settings.

## Figures and Tables

**Table 1:** Demographic characteristics of study participants

**Variable**	**Cases (n=100)**	**Controls (n=200)**	**p-value**
Mean Age (years)	45.2± 14.6	46.8± 15.3	0.35
Gender (M/F)	58/42	118/82	0.78
BMI (kg/m^2^)	24.5± 3.2	24.7± 3.1	0.62

**Table 2:** Distribution of optic nerve disorders in cases

**Disorder**	**Number of Cases (n=100)**	**Percentage (%)**
Optic Neuritis	40	40
Ischemic Optic Neuropathy	25	25
Glaucoma	20	20
Hereditary Optic Neuropathy	10	10
Traumatic Optic Neuropathy	5	5

**Table 3:** VEP parameters in cases and controls

**Parameter**	**Cases Mean± SD**	**Controls Mean± SD**	**p-value**
P100 Latency (ms)	120.5± 10.4	100.3± 5.6	< 0.001*
P100 Amplitude (µV)	5.6± 2.1	8.3± 2.5	< 0.001*
N70 Latency (ms)	70.5± 8.3	65.2± 6.7	0.02*
N155 Latency (ms)	155.8± 12.4	140.3± 10.1	< 0.001*
*Statistically significant

**Table 4:** VEP abnormalities in different optic nerve disorders

**Disorder**	**Prolonged P100 Latency (%)**	**Reduced P100 Amplitude (%)**
Optic Neuritis	90	85
Ischemic Optic Neuropathy	80	70
Glaucoma	60	65
Hereditary Optic Neuropathy	70	60
Traumatic Optic Neuropathy	50	40

**Table 5:** Impact of VEP findings on clinical management

**Management Decision**	**Number of Cases (%)**
Confirmed Diagnosis with VEP	80
Change in Treatment Plan	50
Follow-up with VEP Monitoring	70
No Change in Management	20

**Table 6:** Comparison of VEP parameters by age group in cases

**Age Group (years)**	**P100 Latency (ms) Mean± SD**	**P100 Amplitude (µ V) Mean± SD**	**p-value (Latency)**	**p-value (Amplitude)**
< 20	115.4± 8.9	5.8± 2.0	0.001*	0.05*
21-40	118.6± 9.2	5.5± 2.1	0.002*	0.04*
41-60	122.3± 10.1	5.3± 2.2	< 0.001*	0.02*
> 60	125.8± 12.3	5.0± 2.3	< 0.001*	0.01*

**Table 7:** VEP abnormalities in different genders

**Gender**	**Prolonged P100 Latency (%)**	**Reduced P100 Amplitude (%)**	**p-value (Latency)**	**p-value (Amplitude)**
Male	75	70	0.02*	0.05*
Female	65	60	0.03*	0.04*
*Statistically significant

**Table 8:** Association between duration of symptoms and VEP findings

**Duration of Symptoms (months)**	**Prolonged P100 Latency (%)**	**Reduced P100 Amplitude (%)**	**p-value (Latency)**	**p-value (Amplitude)**
< 1	50	45	0.05*	0.07
03-Jan	70	65	< 0.001*	0.02*
06-Apr	80	75	< 0.001*	< 0.001*
> 6	90	85	< 0.001*	< 0.001*
*Statistically significant

**Table 9:** Predictive value of VEP in differentiating optic nerve disorders

**Disorder**	**Sensitivity (%)**	**Specificity (%)**	**PPV (%)**	**NPV (%)**	**Accuracy (%)**
Optic Neuritis	92	88	90	89	90
Ischemic Optic Neuropathy	85	82	83	84	83
Glaucoma	75	78	76	77	76
Hereditary Optic Neuropathy	80	75	78	77	78
Traumatic Optic Neuropathy	70	72	71	71	71

**Table 10:** Cost-Effectiveness of VEP in Diagnosis and Management

**Parameter**	**VEP Group (n=100)**	**Non-VEP Group (n=100)**	**p-value**
Average Diagnostic Cost (INR)	3000± 500	5000± 800	<0.001*
Average Treatment Cost (INR)	20000± 3500	25000± 4000	<0.001*
Average Time to Diagnosis (days)	15± 4	25± 6	<0.001*
Average Time to Treatment (days)	20± 5	30± 7	<0.001*
Patient Satisfaction Score (1-10)	8.5± 1.2	6.8± 1.5	<0.001*

## References

[1] Petrou PA (2014). Invest Ophthalmol Vis Sci..

[2] Aminoff MJ (1994). J Clin Neurophysiol..

[3] Odom VJ (2010). Doc Ophthalmol..

[4] Kothari R (2016). Scientifica (Cairo)..

[5] Field GD (2007). Annu Rev Neurosci..

[6] Benardete EA, Kaplan E (1999). J Physiol..

[7] Lennie P (1980). Vision Res..

[8] Halliday AM (1972). Lancet..

[9] Shahrokhi F (1978). Arch Neurol..

[10] Cant BR (1978). Electroencephalogr Clin Neurophysiol..

[11] Youl BD (1991). Brain..

[12] Hood DC (2000). Invest Ophthalmol Vis Sci..

[13] Rinalduzzi S (2001). J Neurol Neurosurg Psychiatry..

[14] Holder GE (2004). Eye (Lond)..

[15] Parisi V (2006). Ophthalmology..

[16] Mahapatra AK (1991). Indian J Ophthalmol..

